# Central Nervous System-Endogenous TLR7 and TLR9 Induce Different Immune Responses and Effects on Experimental Autoimmune Encephalomyelitis

**DOI:** 10.3389/fnins.2021.685645

**Published:** 2021-06-15

**Authors:** Ruthe Storgaard Dieu, Vian Wais, Michael Zaucha Sørensen, Joanna Marczynska, Magdalena Dubik, Stephanie Kavan, Mads Thomassen, Mark Burton, Torben Kruse, Reza Khorooshi, Trevor Owens

**Affiliations:** ^1^Department of Neurobiology Research, Institute of Molecular Medicine, University of Southern Denmark, Odense, Denmark; ^2^Department of Clinical Genetics, Odense University Hospital, Institute of Clinical Research, University of Southern Denmark, Odense, Denmark

**Keywords:** toll-like receptor, innate signaling, experimental autoimmune encephalomyelitis, monocytes, Type I interferons

## Abstract

Innate receptors, including Toll like receptors (TLRs), are implicated in pathogenesis of CNS inflammatory diseases such as multiple sclerosis (MS) and its animal model experimental autoimmune encephalomyelitis (EAE). TLR response to pathogens or endogenous signals includes production of immunoregulatory mediators. One of these, interferon (IFN)β, a Type I IFN, plays a protective role in MS and EAE. We have previously shown that intrathecal administration of selected TLR ligands induced IFNβ and infiltration of blood-derived myeloid cells into the central nervous system (CNS), and suppressed EAE in mice. We have now extended these studies to evaluate a potential therapeutic role for CNS-endogenous TLR7 and TLR9. Intrathecal application of Imiquimod (TLR7 ligand) or CpG oligonucleotide (TLR9 ligand) into CNS of otherwise unmanipulated mice induced IFNβ expression, with greater magnitude in response to CpG. CD45+ cells in the meninges were identified as source of IFNβ. Intrathecal CpG induced infiltration of monocytes, neutrophils, CD4+ T cells and NK cells whereas Imiquimod did not recruit blood-derived CD45+ cells. CpG, but not Imiquimod, had a beneficial effect on EAE, when given at time of disease onset. This therapeutic effect of CpG on EAE was not seen in mice lacking the Type I IFN receptor. In mice with EAE treated with CpG, the proportion of monocytes was significantly increased in the CNS. Infiltrating cells were predominantly localized to spinal cord meninges and demyelination was significantly reduced compared to non-treated mice with EAE. Our findings show that TLR7 and TLR9 signaling induce distinct inflammatory responses in the CNS with different outcome in EAE and point to recruitment of blood-derived cells and IFNβ induction as possible mechanistic links between TLR9 stimulation and amelioration of EAE. The protective role of TLR9 signaling in the CNS may have application in treatment of diseases such as MS.

## Introduction

Multiple sclerosis (MS) is an inflammatory demyelinating disease of the central nervous system (CNS). Although there are by now many disease modifying therapies, there is no cure for MS. The concept that endogenous CNS regulatory mechanisms could be accessed and/or amplified to regulate inflammation in MS is attractive. Innate immune pathways represent one mechanism that operates to regulate inflammation. We have shown that stimulation of CNS innate signaling in experimental autoimmune encephalomyelitis (EAE), an animal model for MS, induced recruitment of blood-derived myeloid suppressive cells as well as production of interferon (IFN)β, a Type I IFN (Khorooshi et al., 2015, 2020). The induction of CNS-endogenous IFNβ is of particular interest, since drugs based on this cytokine are used as first-line therapy for MS (McGraw and Lublin, 2013) and several studies have shown a protective role for IFNβ in EAE (Teige et al., 2003; Prinz et al., 2008; Galligan et al., 2010; Melero-Jerez et al., 2019). Type I IFNs are induced by several innate receptors, including endosomal toll-like receptor (TLR)3, 7, and 9, in response to viral and bacterial infections. Accumulating evidence points to agonists for innate receptors as potential therapeutics for MS (Mayo et al., 2012). We have shown that stimulation of TLR3 or TLR9+NOD2 induced CNS-endogenous IFNβ, recruited immune cells including phagocytic myeloid cells to the CNS, and ameliorated EAE (Khorooshi et al., 2015, 2020). The recruited extraparenchymal cells constituted a significant source of IFNβ. These findings suggest CNS endogenous innate signaling as a potential therapeutic route for regulation of neuroinflammation.

We have now extended these studies to evaluate the potential therapeutic effect of TLR7 and TLR9 stimulation within CNS. TLR7 recognizes single-stranded RNA whereas TLR9 recognizes unmethylated CpG DNA (Barton, 2007). Upon ligand recognition both TLR7 and TLR9 signal through MyD88- dependent pathways, leading to induction of a plethora of immunoregulatory mediators, including IFNβ (Kaisho and Tanaka, 2008). Responses to intracerebroventricular delivery of TLR7 and TLR9 ligands in neonatal mice differed (Butchi et al., 2011). TLR9 stimulation induced a more potent inflammatory response with elevated levels of several cytokines and chemokines that were correlated with recruitment of cells into the CNS whereas TLR7 stimulation induced a limited cytokine/chemokine response (including IFNβ), without leukocyte recruitment (Butchi et al., 2011). Peripheral administration of a synthetic TLR7 ligand (Imiquimod) or the TLR9 ligand CpG to mice that were immunized for EAE ameliorated subsequent symptoms (O’Brien et al., 2010; Longhini et al., 2014; Crooks et al., 2017). Whether such treatment has clinical effect when given after disease onset was not addressed. It is also unclear whether engagement of TLR7 or TLR9 within the CNS can impact EAE and underlying mechanisms have not been studied. We hypothesized that central stimulation of TLR7 and TLR9 would have protective effect on EAE by mechanisms involving both recruitment of blood-derived cells and induction of Type I IFNs. Our findings show that intrathecal treatment with CpG, but not Imiquimod, induced a strong IFNβ response and infiltration of extraparenchymal blood-derived cells, and suppressed EAE which was dependent on Type I IFN signaling. We link the CpG protective action to its ability to recruit monocytes into the CNS and potent induction of IFNβ.

## Materials and Methods

### Mice

Female albino (C57BL/6-Tyr^*c–2J*^) IFNβ^+/Δβ–luc^ mice (IFNβ/luciferase reporter mice) (Lienenklaus et al., 2009), Interferon-α receptor 1–deficient (IFNAR1-KO) mice (C57BL/6 background) and Yellow Fluorescent Protein (YFP) (IFN-β^*mob/mob*^) IFNβ knock-in mice (Scheu et al., 2008) were all bred and housed in the Biomedical Laboratory, University of Denmark. Female C57BL/6j mice were purchased from Taconic Europe A/S (Lille Skensved, Denmark). All animal experiments were conducted in accordance with Danish national ethical committee (Animal Experiments inspectorate under Danish Ministry of Food, Agriculture and Fisheries, The Danish Veterinary and Food Administration, approval identification number: 2020-15-0201-00652).

### Experimental Autoimmune Encephalomyelitis Induction

Female C57BL/6 and IFNAR1-KO mice between age 8–12 weeks were immunized by subcutaneous injection in the flanks with 100 μl emulsion containing 100 μg myelin oligodendrocyte glycoprotein (MOG) p35–55 (sequence MEVGWYRSPFSRVVHLYRNGK, TAG) and Complete Freund’s Adjuvant (BD Biosciences, Denmark) with 200 μg heat-inactivated *Mycobacterium tuberculosis* (BD Biosciences). Mice received an intraperitoneal (ip) injection of *Bordetella pertussis* toxin (300 ng, Sigma-Aldrich) at the time of immunization and 1-day post- immunization. Mice were then monitored daily for loss of body weight and EAE symptoms. The EAE grades were defined as follows: grade 0, no signs of disease; grade 1, weak or hooked tail; grade 2, floppy tail indicating complete loss of tonus in tail; grade 3, floppy tail and hind limb paresis, grade 4: floppy tail and unilateral hind limb paralysis; grade 5, floppy tail and bilateral hind limb paralysis. Due to ethical reasons, mice were sacrificed if they reached grade 5 or if hind limb paralysis persisted for 2 days. Clinical scores of euthanised grade 5 animals were retained in graphic representation of disease progression (Supplementary Figures 1–3).

### Intrathecal Injection

Mice were anesthetized by inhalation of 2–4% isoflurane (Abbott Laboratories). They received Temgesic (Reckitt Benckiser Pharmaceuticals Ltd.) in isotonic sterile saline (9 mg/ml NaCl, Fresenius Kabi) for pain relief and the back of the neck was shaved. A 30-gauge needle (bent 55° with a 2 mm tip) attached to a 50 μl Hamilton syringe was used to perform intrathecal injection into the cisterna magna, for administration into the cerebrospinal fluid. After the injections, mice received subcutaneous injection of 1 ml of isotonic sterile saline for prevention of dehydration.

Mice were intrathecally injected with CpG (ODN 1585, class A, Invivogen), Imiquimod (R837, Invivogen). Control mice received vehicle alone, either phosphate buffered saline (PBS) or 1 × Hanks balanced salt solutions (HBSS) (Gibco).

In trial experiments evaluated by *in vivo* imaging of luciferase reporter mice, IFNβ expression in response to CpG and Imiquimod was dose-dependent (not shown). Based on the IFNβ expression, the optimal dose for CpG and Imiquimod was determined to be 10 and 50 μg, respectively, and these were used throughout the study.

### *In vivo* Imaging

*In vivo* imaging of luciferase activity as a reporter for IFNβ was performed by injecting D-luciferin (150 mg/kg) intraperitoneally to IFN-β+/Δβ-luc mice 10 min prior to image capture. Mice were then anesthetized with 2–4% isoflurane and monitored using an IVIS 200 imaging system (CaliperLS) (DaMBIC). Photon flux was quantified using Living Image 4.4 software (CaliperLS).

### Tissue Processing

Mice were euthanized with an overdose of sodium pentobarbital (100 mg/kg, Glostrup Hospital) and perfused with ice-cold PBS. For reverse transcriptase-quantitative polymerase chain reaction (RT-qPCR), brains and spinal cords were placed in 0.5 ml TriZol Reagent (Ambion) and stored at −80°C until RNA extraction. For flow cytometry, CNS tissue was placed in ice-cold PBS. For histology, brains and spinal cords were post-fixed with 4% paraformaldehyde (PFA), immersed in 30% sucrose in PBS, then frozen embedded in Killik cryostat embedding medium (Bio-Optica, Milano, Italy) and 16 μm thick tissue sections were cut on a cryostat (Leica).

### Flow Cytometry

A single cell suspension was obtained by chopping the tissue and then forcing the dissociated CNS tissue through a 70 μm cell strainer (Falcon, United States) in HBSS supplemented with 2% fetal bovine serum (FBS). Myelin clearance was obtained by centrifugation on 37% Percoll (GE Healthcare Bio-Science AB) followed by aspiration of the myelin layer. Cells were incubated in blocking solution containing HBSS, 2% FBS, anti-CD16/32 antibody (Clone 2.4G2, BD Biosciences), Syrian hamster IgG (50 μg/ml, Jackson ImmunoResearch Laboratories Inc.) and 0.01% sodium azide, and then labeled with fluorophore-conjugated antibodies (BioLegend): anti-CD45 (clone 30-F11), CD11b (M1/70), F4/80 (BM8), GR-1 (RB6-8C5), NK1.1 (PK136), CD4 (GK1.5), and TCRβ (H57-597). Fluorescence data were acquired on an LSRII flow cytometer (BD Biosciences) with FACSDiva software (BD Biosciences) and analyzed with Flowlogic (Inivai Technologies).

### Histology

To identify the localization and cellular source of IFNβ, brain sections from YFP/IFNβ reporter mice that had received CpG or Imiquimod by intrathecal injection were incubated in blocking solution containing PBST and 3% bovine serum albumin (BSA), followed by incubation with the following primary antibodies: polyclonal rabbit anti-green fluorescent protein (GFP) (ab6556; Abcam) (Scheu et al., 2008), PE-conjugated rat anti-mouse CD45 (#103106, Biolegend). Sections were then washed with PBST and incubated with biotinylated goat anti rabbit IgG (H+L) (#64256, Abcam), followed by incubation with streptavidin–horseradish peroxidase (RPN1231V, GE Healthcare), washed in PBS and GFP staining was developed using the TSA^TM^ System (PerkinElmer) according to the manufacturer’s instructions. Nuclei were visualized by 4′,6-diamidino-2-phenylindole (Dapi) staining and the sections were mounted with gelvatol (Khorooshi et al., 2015). The specificity of primary antibody was verified as described previously (Khorooshi et al., 2020). Hematoxylin and eosin (H&E) and anti-MOG staining were performed as described previously (Khorooshi et al., 2015; Wlodarczyk et al., 2018). Images were acquired using an Olympus DP71 digital camera mounted on an Olympus BX51 microscope (Olympus) or with an Olympus FV1000MPE Confocal and Multiphoton Laser Scanning Microscope, Danish Molecular Biomedical Imaging Center (DaMBIC), University of Southern Denmark. Images were acquired using 4× and 10× objectives and combined using Adobe Photoshop CS3 (Adobe Systems Denmark A/S).

### RNA Isolation and Quantitative RT-PCR

RNA extraction from brains and spinal cords was performed as described previously (Khorooshi et al., 2020). RNA concentration was measured on a NanoDrop spectrophotometer (Nanodrop ND-1000 Spectrophotometer, Thermo Scientific) and 1 μg RNA was converted into cDNA using a high-capacity cDNA reverse transcription kit (Applied Biosystems). qRT-PCR was performed using an ABI Prism 7300 sequence detection system (Applied Biosystems), using primers and probes described earlier (Khorooshi et al., 2015). For normalization of gene expression 18S rRNA was used. Ct values were determined, and results are presented as fold change or gene of interest relative to 18S rRNA (2^Δ*CT*^ method). If Ct signal was not detected, the Ct value relative to 18S rRNA was set to 0.

### RNA Sequencing

For RNA sequencing (RNAseq), RNA was isolated from brains as described in the previous section, and the quality of RNA was checked using an Agilent bioanalyzer. The sequence libraries were prepared using an Illumina Truseq Stranded mRNA sample preparation kit (Illumina). RNA sequencing was performed with Illumina NextSeq sequencer using 2 × 75 bp paired end reads. The raw data were then analyzed as described previously (Khorooshi et al., 2020).

### Statistical Analysis

The Rout test (*Q* = 1) was used to estimate significant outliers that were removed before further statistical testing. Data were tested for normal distribution and analyzed by two tailed non-parametric Student’s *T* Test followed by Mann–Whitney *U*-test. For statistical evaluation of more than two groups one-way ANOVA with Bonferroni’s multiple comparisons test or with multiple comparisons uncorrected Fisher’s LSD test were used. Fisher’s exact test was used for statistical evaluation on mice with EAE following intrathecal CpG or Imiquimod treatment. All statistical analysis was performed using GraphPad Prism version 6 (Graphpad Software Inc.). Results are presented as means ± SD. Values of *p* < 0.05 were considered as significant.

## Results

### Intrathecal CpG and Imiquimod Induced Overlapping but Distinct Immune Response in the CNS

**FIGURE 1:**
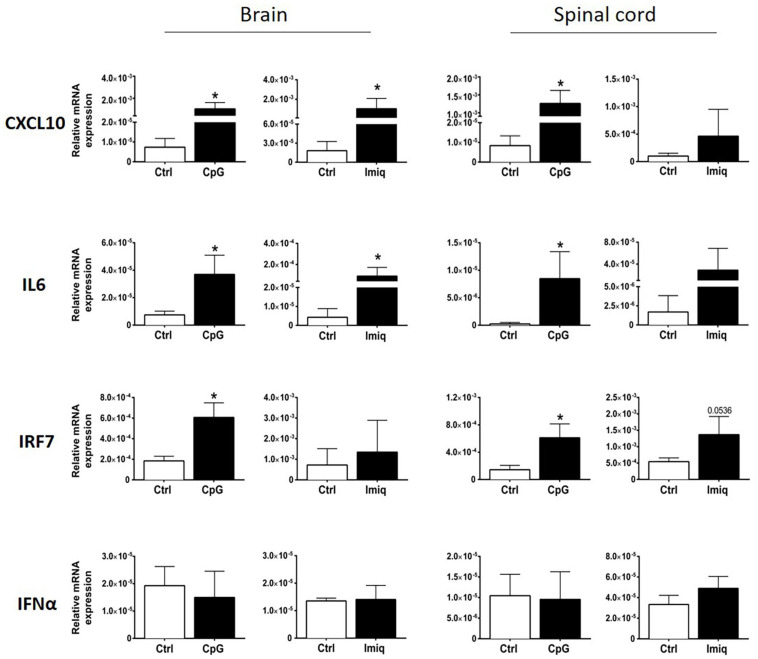
Intrathecal CpG and Imiquimod induced overlapping but distinct immune response in the central nervous system (CNS). mRNA levels of CXCL10, IL6, IRF7, and IFNα (*n* = 3–6 per group) in brains and spinal cords were analyzed 4 h post intrathecal injection of CpG and Imiquimod (Imiq) into healthy mice. Results were analyzed using the two-tailed Mann–Whitney *U*-test. Data are presented as means ± SD. *p < 0.05.

We examined induction of cytokines and chemokines in brains and spinal cords of healthy mice 4 h post-intrathecal injection of Imiquimod and CpG. Imiquimod was used for TLR7 stimulation because the induction of neuroinflammatory responses by Imiquimod is dependent on TLR7 (Butchi et al., 2008). Significant levels of CXCL10 and IL6 were induced in the brain by both intrathecal CpG and Imiquimod (Figure 1), whereas significant induction in the spinal cord was only triggered by intrathecal CpG (Figure 1). Intrathecal CpG but not Imiquimod induced significant increase of IRF7 mRNA levels in both brain and in spinal cord. Levels of IFNα mRNA did not change in response to intrathecal CpG or Imiquimod (Figure 1).

### Intrathecal CpG Induced Higher Levels of IFNβ in the CNS in Comparison to Imiquimod

**FIGURE 2:**
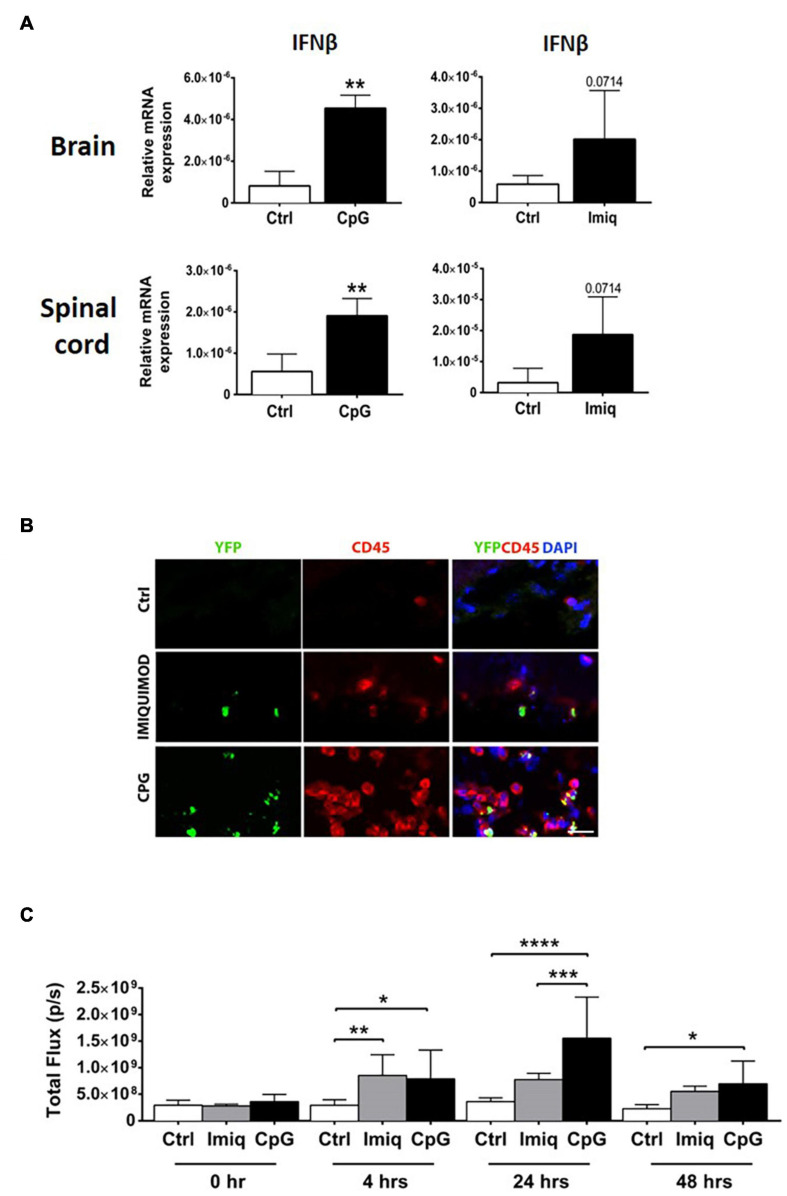
Intrathecal CpG induced higher levels of IFNβ in the CNS in comparison to Imiquimod. Brains and spinal cords were analyzed 4 h post intrathecal injection of CpG and Imiquimod (Imiq) into healthy mice. **(A)** Bar graphs show mRNA levels of IFNβ (*n* = 3–6 per group). Results were analyzed using the two-tailed Mann–Whitney *U*-test. **(B)** Representative micrographs of brain sections from control mice and mice that received intrathecal Imiquimod or CpG 4 h earlier. Co-localization of IFNβ/YFP+ (green) and CD45+ (red) cells in mice is shown. Nuclei were stained with DAPI (blue). Scale bar: 20 μM. **(C)** IFNβ/luciferase reporter mice received either intrathecal CpG or Imiquimod and *in vivo* imaging measured the level of IFNβ/luciferase at 0, 4, 24, and 48 h post injection (*n* = 4–7 per group). Results were analyzed using one-way ANOVA with multiple comparisons uncorrected Fisher’s LSD test. Data are presented as means ± SD. *p < 0.05; **p < 0.01; ***p < 0.001; ****p < 0.0001.

We examined induction of IFNβ in the brains and spinal cords of healthy mice 4 h post-intrathecal injection of CpG and Imiquimod. Levels of IFNβ mRNA in the CNS were significantly induced in response to CpG and were elevated (not to significance) in response to Imiquimod (Figure 2A).

To investigate the cellular source of IFNβ, IFNβ/YFP knockin (IFNβmob/mob) mice were used (Scheu et al., 2008). IFNβ/YFP+ expressing cells that had rounded morphology were distributed in leptomeningeal space (Figure 2B). Double immunostaining showed IFNβ co-localization with CD45+ cells. IFNβ/YFP+ CD45 expressing cells were more abundant in mice that had received CpG, compared to Imiquimod-treated or control mice (Figure 2B).

We further examined induction of IFNβ using mice that express luciferase under control of an IFNβ promotor (Lienenklaus et al., 2009). Luciferase activity was measured at 4, 24, and 48 h post-intrathecal injection of CpG or Imiquimod. *In vivo* imaging showed that both Imiquimod and CpG induced a similar significant increase in IFNβ response at 4 h compared to control mice (Figure 2C). The expression of IFNβ in response to intrathecal Imiquimod remain elevated although reduced at 24 and 48 h post injection (Figure 2C). In contrast, IFNβ response to intrathecal CpG was significantly increased at 24 compared to Imiquimod and was reduced but still at significant levels at 48 h (Figure 2C). As expected, intrathecal injection of vehicle alone did not induce IFNβ at any timepoint (Figure 2C).

### Intrathecal CpG Induced Cytokine and Chemokine Gene Expression and Cell Recruitment in the Healthy CNS

**FIGURE 3:**
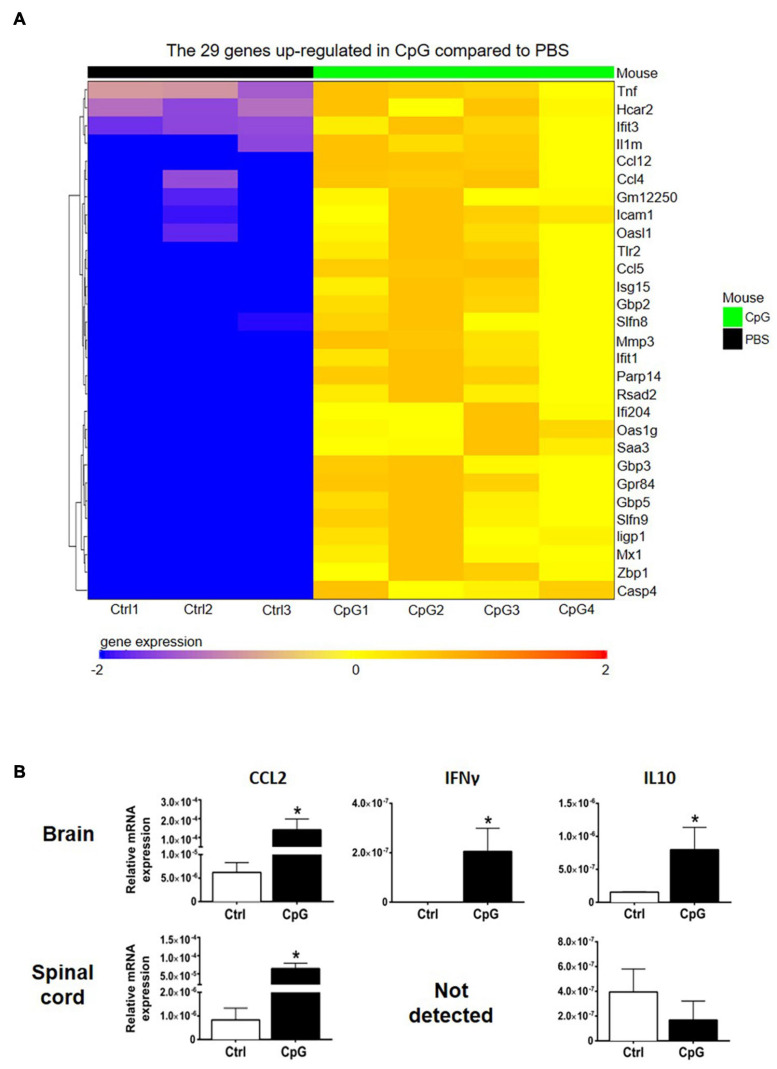
Intrathecal CpG induced inflammation-associated cytokines and chemokines. To investigate how intrathecal CpG influences CNS inflammatory programs in healthy mice, expression of inflammation-associated mediators was analyzed by RNAseq and RT-pPCR. **(A)** Heatmap of RNAseq analysis of CpG-induced CNS. Intrathecal CpG induced significant upregulation of 29 genes in the brain (LogFC ≥ 2 and adjusted *p*-value ≤ 0.05). These genes include type I IFN response genes as well as chemokines. **(B)** Bar graphs show levels of CCL2, IFNγ, and IL10 mRNA in brains and spinal cords 4 h post injection (*n* = 3–5 per group). Results were analyzed using the two-tailed Mann–Whitney *U*-test. Data are presented as means ± SD. *p < 0.05.

RNA sequencing analysis showed upregulation of several chemokines in CNS of CpG-treated mice (Figure 3A). In addition, we found upregulation of CCL2, IFNγ, and IL10 mRNA in the CNS by RT-qPCR (Figure 3B).

**FIGURE 4:**
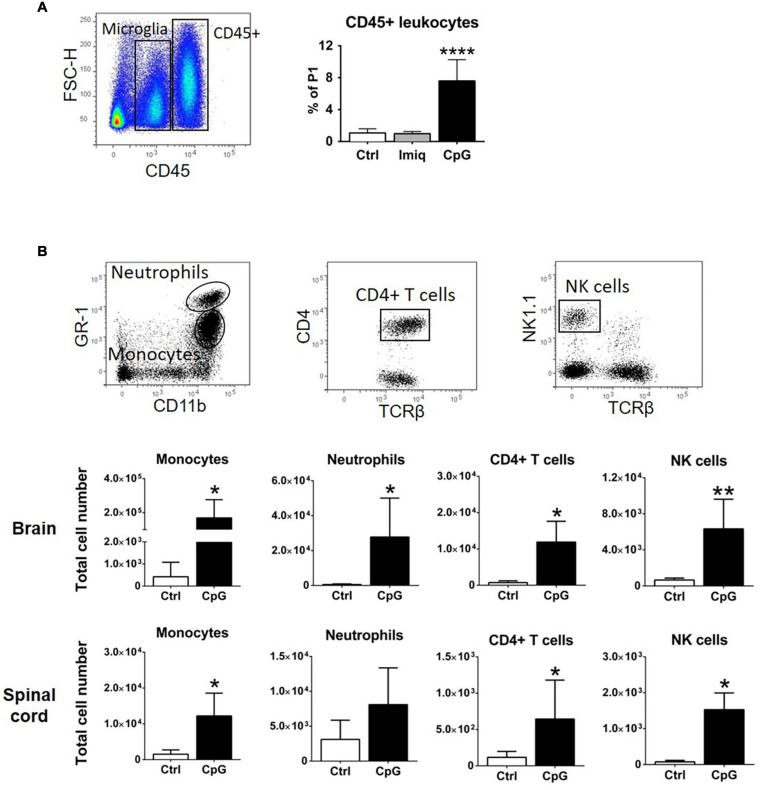
Intrathecal CpG induced recruitment of immune cells to the healthy CNS. **(A)** Flow cytometric gating strategy used to distinguish CD45+ leukocytes from CD45dim microglia. The bar graph shows that intrathecal CpG but not Imiquimod (Imiq) significantly increased the number of CD45+ cells in the CNS (pooled brain and spinal cord) 4 h post injection (*n* = 5–7 per group). Results were analyzed using one-way ANOVA with Bonferroni’s multiple comparisons test. **(B)** Representative flow cytometry profiles showing gating strategies to distinguish monocytes (CD45^*hi*^CD11b^*hi*^GR1^*low/*–^ F4/80^+^), neutrophils (CD45^*hi*^CD11b^*hi*^GR1^*hi*^F4/80^–^), CD4+ T cells (CD45^*hi*^CD11b^*low/*–^ TCRβ^+^CD4^+^), and NK cells (CD45^*hi*^CD11b^*low/*–^ TCRβ^–^ NK1.1^+^). Bar graphs show numbers of cells 24 h post injection in brains and spinal cords (*n* = 4–6 per group). Results were analyzed using the two-tailed Mann–Whitney *U*-test. Data are presented as means ± SD. *p < 0.05; **p < 0.01; ****p < 0.0001.

We have previously shown that intrathecal Poly-I:C and MIS416, a TLR3 agonist and TLR9+NOD2 bispecific microparticle, respectively, induced recruitment of CD45+ cells into the CNS (Khorooshi et al., 2015, 2020). Flow cytometry showed significant increase in CD45+ cells in the CNS of CpG-treated healthy mice compared to Imiquimod-treated or control animals 4 h post injection (Figure 4A). We analyzed the phenotype of the CD45+ cells that were recruited 24 h post intrathecal CpG (Figure 4B). CpG induced recruitment of monocytes (CD45^*hi*^CD11b^*hi*^GR1^*low/*–^F4/80^+^), neutrophils (CD45^*hi*^CD11b^*hi*^GR1^*hi*^F4/80^–^), CD4+ T cells (CD45^*hi*^CD11b^*low/–*^TCRβ^+^CD4^+^), as well as NK cells (CD45^*hi*^CD11b^*low/*–^TCRβ^–^NK1.1^+^) into the brain and spinal cord (Figure 4B).

### Intrathecal CpG, but Not Imiquimod, Suppressed EAE in an IFNAR-Dependent Manner

**FIGURE 5:**
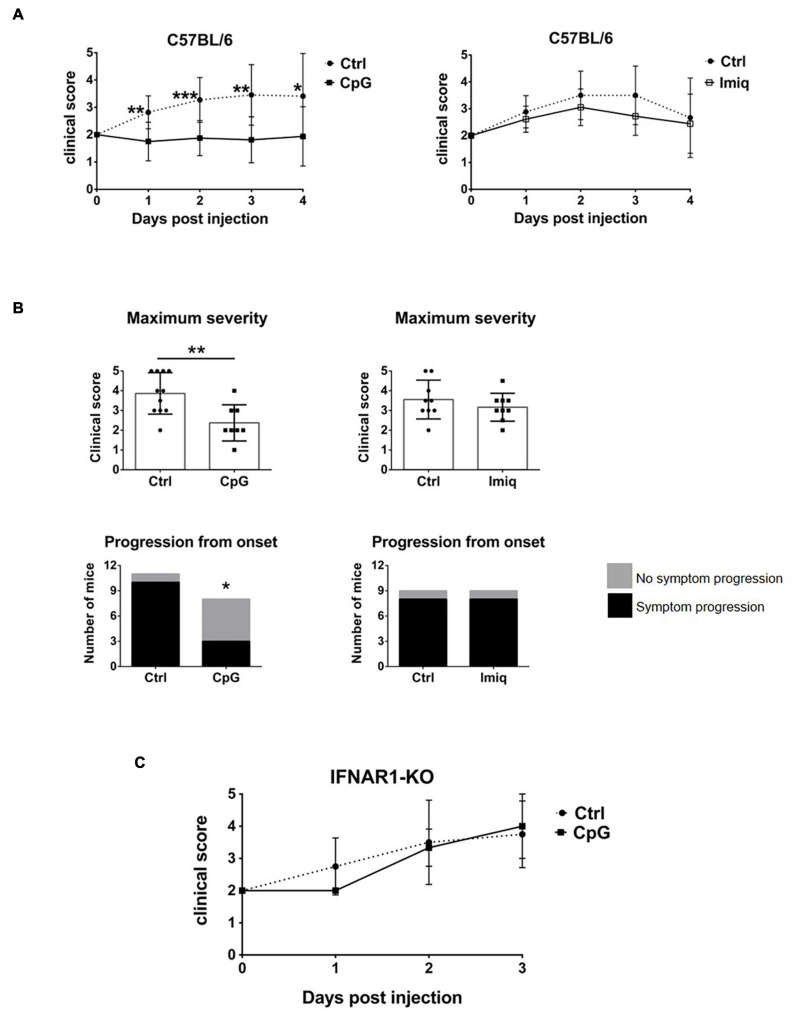
Intrathecal CpG, but not Imiquimod, suppressed EAE in an IFNAR-dependent manner. Mice were immunized with MOG35-55 to induce EAE and at disease onset (day 0) they received intrathecal CpG or Imiquimod (Imiq). Symptoms were scored daily until day 4. **(A)** Clinical scores of C57BL/6 mice with EAE treated with intrathecal CpG or Imiquimod (*n* = 8–11 per group). Results were analyzed using the two-tailed Mann–Whitney *U*-test (comparing two groups at a given day). **(B)** Maximum severity based on the highest clinical score of each mouse and clinical scores of mice whose symptoms progressed or not after treatment at day 0. CpG treatment significantly reduced EAE severity as well as the number of mice that progressed after onset (*n* = 8–11 per group). Results were analyzed using the two-tailed Mann–Whitney *U*-test (top graphs) and Fisher’s exact test (bottom graphs). **(C)** IFNAR1-KO mice with EAE were treated with intrathecal CpG (*n* = 3–8 per group). Data are presented as means ± SD. *p < 0.05; **p < 0.01; ***p < 0.001.

TLR7 and 9 are both expressed in the CNS, but whether their stimulation within the CNS plays a role in the regulation of EAE has not been studied. We therefore examined whether intrathecal CpG or Imiquimod would affect EAE. Mice were immunized to induce EAE and at the onset of disease (day 0, defined as when mice first showed symptoms), they received a single intrathecal injection of CpG or Imiquimod. Disease progression was followed for 4 days. Disease severity was reduced in mice that received intrathecal CpG, whereas intrathecal Imiquimod treatment had no effect (Figure 5A), even when mice were treated with a repeated intrathecal injection at day 2. Supplementary Figures 1–3 show clinical scores over the 4-days period for individual mice. The maximum clinical score of mice treated with intrathecal CpG was significantly reduced whereas it did not change in response to Imiquimod treatment (Figure 5B). A significantly higher number of mice did not progress by increased severity of symptoms in response to CpG treatment than in response to Imiquimod or in controls (Figure 5B).

Suppression of EAE by intrathecal innate receptor ligands has been shown to be dependent on Type I IFN signaling (Khorooshi et al., 2015, 2020). To assess whether this was also the case for EAE suppression by CpG, IFNAR1-deficient mice were treated with intrathecal CpG at the onset of symptoms. Intrathecal CpG had no effect on EAE progression in IFNAR1 deficient mice (Figure 5C), indicating that IFNAR signaling is also required for the EAE-suppressive effect of CpG action.

### Intrathecal CpG Induced Recruitment of Monocytes to the CNS and Reduced Demyelination in EAE

**FIGURE 6:**
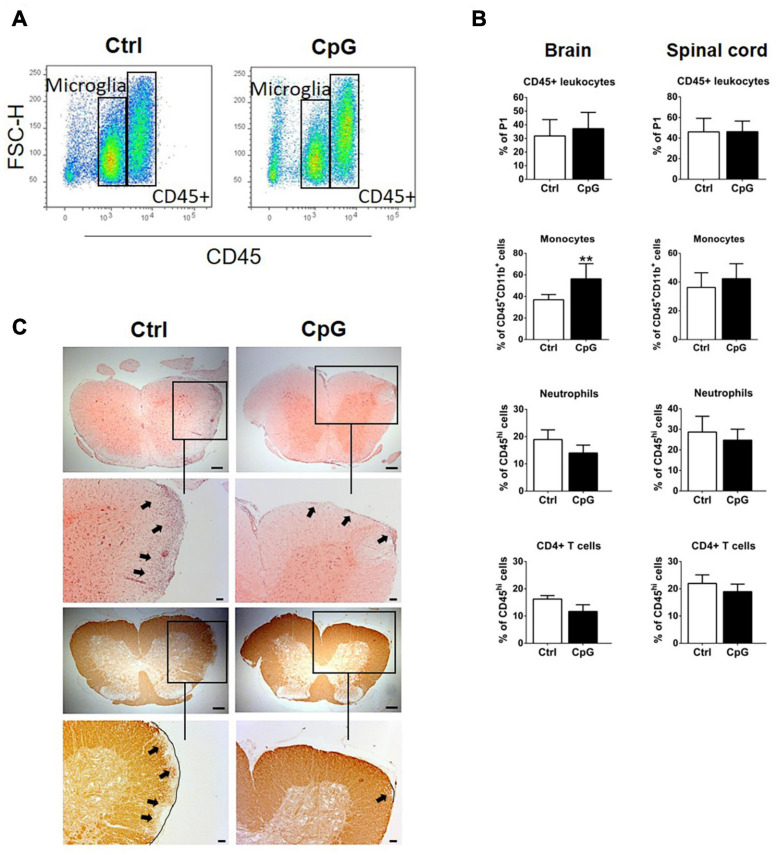
Intrathecal CpG induced recruitment of monocytes to the CNS and reduced demyelination in experimental autoimmune encephalomyelitis (EAE). **(A)** Flow cytometry profiles of mice with EAE treated with intrathecal CpG or control (ctrl). CD45high leukocyte populations are distinguished from CD45dim microglia. **(B)** Quantitative flow cytometric analysis comparing the percentage of CD45+ leukocytes, (CD45^*hi*^CD11b^*hi*^GR1^*low/*–^ F4/80^+^) monocytes, neutrophils (CD45^*hi*^CD11b^*hi*^GR1^*hi*^F4/80^–^), and CD4+ T cells (CD45^*hi*^CD11b^*low/*–^ TCRβ^+^CD4^+^) (*n* = 6–8 per group). Results were analyzed using the two-tailed Mann–Whitney *U*-test. **(C)** Representative images of spinal cord sections of mice with EAE stained with H&E and anti-MOG antibody. Boxes show selected areas of the spinal cord sections with higher magnification. Arrows highlight infiltration or loss of MOG. Scale bars: 200 and 100 μm. Data are presented as means ± SD. **p < 0.01.

We used flow cytometry to ask whether and how CpG influenced infiltration of immune cells into the brain and spinal cord during EAE. Percentages of CD45+ cells were not different between control and CpG treated mice 24 h after receiving intrathecal injection (Figures 6A,B). Further analysis revealed that infiltrating monocytes were significantly increased in CpG-treated versus EAE-control mice, whereas T cells and neutrophils showed tendencies to reduction (Figure 6B). H&E staining localized infiltrating cells in the parenchyma of spinal cord in control EAE mice (Figure 6C). By contrast, infiltration in spinal cord sections of CpG treated mice was predominantly observed in the meninges. Staining with anti-MOG revealed loss of MOG in corresponding areas in control mice, which was reduced after CpG treatment.

## Discussion

In this present study, we have shown that stimulation of TLR7 and TLR9 in the CNS resulted in overlapping but distinct induction of several chemokines, IFNβ and IFNAR-dependent genes as well as pro- and anti-inflammatory cytokines. CNS-infiltrating leukocytes induced by CpG but not by Imiquimod were identified as monocytes, neutrophils, CD4+ T cells and NK cells. Only intrathecal CpG suppressed EAE progression and this was IFNAR-dependent. Monocytes were selectively increased by CpG treatment in CNS of mice with EAE. Infiltrates in spinal cord of CpG-treated mice with EAE were predominately found in the meninges, unlike parenchymal infiltrates in EAE controls. Demyelination was reduced by CpG treatment.

Both TLR7 and TLR9 stimulation induced IFNβ but to different extent, CpG induction being more pronounced. Whether this reflects a true distinction in signaling or different pharmacokinetics was not determined. CpG also induced the anti-inflammatory cytokine IL10 in the CNS. IL10 and IFNβ may contribute to the anti-inflammatory environment in the CNS and both are known to play a role in regulation of EAE (Bettelli et al., 1998; Khorooshi et al., 2015). The class A CpG used in this study strongly induces IFNβ through IRF7 signaling but is a relatively weak stimulator of TLR9-dependent NF-κB signaling (Krug et al., 2001; Guiducci et al., 2006).

The fact that Imiquimod induced a 10-fold increase in IL6 mRNA expression and CpG only induced a threefold induction (Figure 1) is consistent with TLR7 being a stronger stimulator of the NF-κB pathway (Libermann and Baltimore, 1990; Brasier, 2010). It has been shown that activation of NF-κB, particularly in microglia and astrocytes, promotes EAE development, and NF-κB-deficient mice are resistant to EAE (Hilliard et al., 1999; Yue et al., 2018). Furthermore, mice that lack IL6 expression in astrocytes showed amelioration of EAE symptoms (Erta et al., 2016). Whether these differences in responses to intrathecal CpG or Imiquimod are responsible for their distinct effect on EAE remains unclear.

We found that CXCL10 was induced in response to both Imiquimod and CpG. However, in contrast to Imiquimod, CpG induced recruitment of CD45+ cells into the CNS that were a source of IFNβ. Cellular infiltration to the CNS in response to intracerebroventricular injection of CpG but not Imiquimod was also reported in neonatal mice (Butchi et al., 2011). That study also showed similar CXCL10 expression by TLR7 and TLR9 ligands, but CCL2 and other chemokines that were also induced in response to CpG remained similar to control levels in response to Imiquimod. Our findings of different infiltration are in broad agreement with this study. Whether differential chemokine responses contribute to this remains to be determined. The phenotypes of cells recruited by CpG to the CNS included monocytes, neutrophils, CD4+ T cells and NK cells, consistent with chemokine profiles. It is likely that differential leukocyte infiltration contributed to the differential IFNβ response, although this has not been rigorously examined.

Several studies have focused on the role of Imiquimod and CpG in EAE after peripheral administration. Intraperitoneal administration of Imiquimod prior to disease onset induced IFNβ and suppressed disease severity in a chronic model of EAE (O’Brien et al., 2010). We did not find that Imiquimod had suppressive effect against EAE, when intrathecal treatment was given at disease onset. Also, EAE was not exacerbated by intrathecal Imiquimod, contradicting a study in TLR7 knockout mice that suggested TLR7 signaling exacerbates EAE (Lalive et al., 2014).

Peripheral treatment of mice with CpG before disease onset significantly reduced EAE severity (Smolianov et al., 2011; Longhini et al., 2014; Crooks et al., 2017). Also, unmethylated CpG motifs used as adjuvant in vaccination against EAE in rats had protective properties (Lobell et al., 1999). However, studies in TLR9-deficient mice have generated contradictory findings (Prinz et al., 2006; Marta, 2009) and activation of TLR9 has been suggested to exacerbate pathogenesis in EAE (Ichikawa et al., 2002). Our finding that intrathecal administration of CpG suppressed EAE supports a protective role for TLR9 in the CNS.

The ability of CpG to ameliorate EAE symptoms and to trigger recruitment of CD45+ cells into the CNS as well as induce a robust IFNβ response is consistent with our previous studies (Khorooshi et al., 2015, 2020). There we showed that intrathecally-delivered Poly-I:C and a bispecific microparticle (MIS416) induced both recruitment of blood-derived immune cells to the CNS as well as an IFNβ response and led to suppression of EAE (Khorooshi et al., 2020). Together these findings suggest that both IFNβ response and immune cell recruitment to the CNS contribute to the endogenous anti-inflammatory responses that direct a protective effect on EAE pathology. We have recently showed that neutrophils recruited in response to CNS innate stimulation by MIS416 play a protective role in EAE (Khorooshi et al., 2020). These cells were also a source of IFNβ. Here, we observed that the proportion of monocytes was increased in response to intrathecal CpG treatment in mice with EAE and recruited CD45+ cells were a source of IFNβ. We also showed that intrathecal CpG treatment induced Type I IFN associated genes in the CNS, as well as chemokines that are important for directing monocyte migration.

A recent study showed that protection against EAE progression was mediated by IFNβ-induced myeloid derived-suppressor cells (Melero-Jerez et al., 2019). Resembling our findings, they also showed restricted parenchymal infiltration in protected mice. In addition, they found a higher number of arginase-1+ myeloid-derived suppressor cells among infiltrating cells in IFNβ-treated mice and linked them to augmenting T cell apoptosis. We found significantly elevated levels of Arginase1 mRNA in the spinal cord of healthy mice 24 h post intrathecal CpG (Supplementary Figure 4) indicating a possible role for CpG-induced Arginase1. However, whether CpG treatment in EAE works through similar mechanisms to limit parenchymal infiltration and T cell response requires further studies.

CD11b^+^Ly6C^*high*^ monocytes have been shown to be responsible for suppressing T cells in EAE (Zhu et al., 2007). Intrathecal CpG treatment into mice with EAE induced IFNβ and led to an increase in the number of CD45^*hi*^CD11b^*hi*^GR1^*low/–*^F4/80^+^ monocytes–further work is required to reveal if they mediate the protective action of CpG. Our recent study of effect of a TLR9+NOD2 bispecific microparticle showed that protective neutrophils that were recruited suppressed EAE, whereas blood- derived monocytes did not protect against EAE progression (Khorooshi et al., 2020). Despite that numbers of neutrophils were not altered in response to CpG treatment in EAE in the present study, they may potentially have contributed to protection.

**FIGURE 7:**
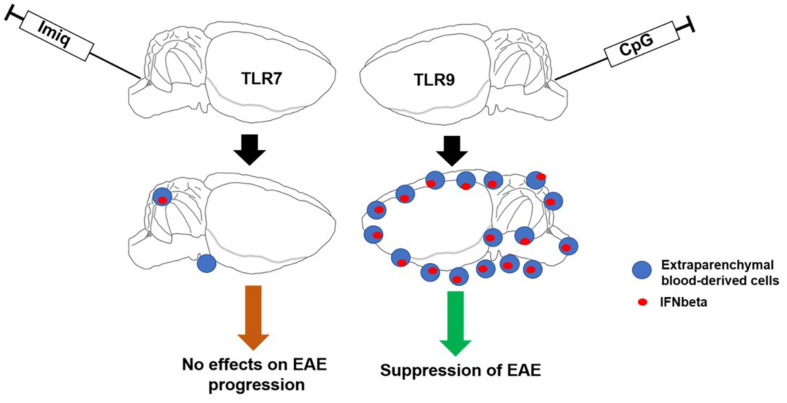
Schematic illustrating the major findings from this study. Activation of TLR9 signaling pathways within the CNS induces IFNβ production and recruitment of peripheral cells which contribute to suppression of EAE.

In conclusion, selective activation of TLR signaling pathways within the CNS that induces IFNβ production and recruitment of peripheral cells may have therapeutic potential in inflammatory demyelinating diseases such as MS (Figure 7).

## Data Availability Statement

The datasets presented in this study can be found in online repositories. The names of the repository/repositories and accession number(s) can be found below: https://www.ncbi.nlm.nih.gov/geo/query/acc.cgi?acc=GSE172083.

## Ethics Statement

The animal study was reviewed and approved by Animal Experiments inspectorate under Danish Ministry of Food, Agriculture and Fisheries, The Danish Veterinary and Food Administration, approval identification number: 2020-15-0201-00652.

## Author Contributions

RK, RD, and TO designed the experiments and wrote the manuscript. RD, RK, VW, MS, JM, and MD performed the experiments and analyzed data. SK, MT, MB, and TK generated and analyzed RNAseq data. All authors contributed to the article and approved the submitted version.

## Conflict of Interest

The authors declare that the research was conducted in the absence of any commercial or financial relationships that could be construed as a potential conflict of interest.

## Supplementary Material

Supplementary Figure 1 Clinical scores of individual C57BL/6 mice with EAE that received CpG. Individual clinical scores of mice with EAE treated with CpG or vehicle (Ctrl). C57BL/6 mice were immunized with MOG35-55. At first sign of disease (day 0), mice received intrathecal injection of CpG or vehicle. A cross indicates the timepoint where a mouse was euthanized.

Supplementary Figure 2 Clinical scores of individual C57BL/6 mice with EAE that received Imiquimod. Individual clinical scores of mice with EAE treated with Imiquimod (Imiq) or vehicle (Ctrl). C57BL/6 mice were immunized with MOG35-55. At first sign of disease (day 0), mice received intrathecal injection of Imiquimod or vehicle. A cross indicates the timepoint where a mouse was euthanized.

Supplementary Figure 3 Clinical scores of individual IFNAR1-KO mice with EAE that received CpG. Individual clinical scores of mice with EAE treated with CpG or vehicle (Ctrl). C57BL/6 mice were immunized with MOG35-55. At first sign of disease (day 0), mice received intrathecal injection of CpG or vehicle.

Supplementary Figure 4 Intrathecal CpG induces Arginase1. Brains and spinal cords were analyzed 4 and 24 h post intrathecal injection of CpG and Vehicle (Ctrl) into healthy C57BL/6 mice. Bar graphs show mRNA levels of Arginase1 (Arg1) (*n* = 3–5 per group). Results were analyzed using the two-tailed Mann–Whitney *U*-test. Data are presented as means ± SD. ^∗^*p* < 0.05.
