# Supplementary figures and images for: Central Nervous System-Endogenous TLR7 and TLR9 Induce Different Immune Responses and Effects on Experimental Autoimmune Encephalomyelitis
Source: Front Neurosci. 2021 Jun 15;15:685645. doi: 10.3389/fnins.2021.685645 (PMC8241214; doi:10.3389/fnins.2021.685645)

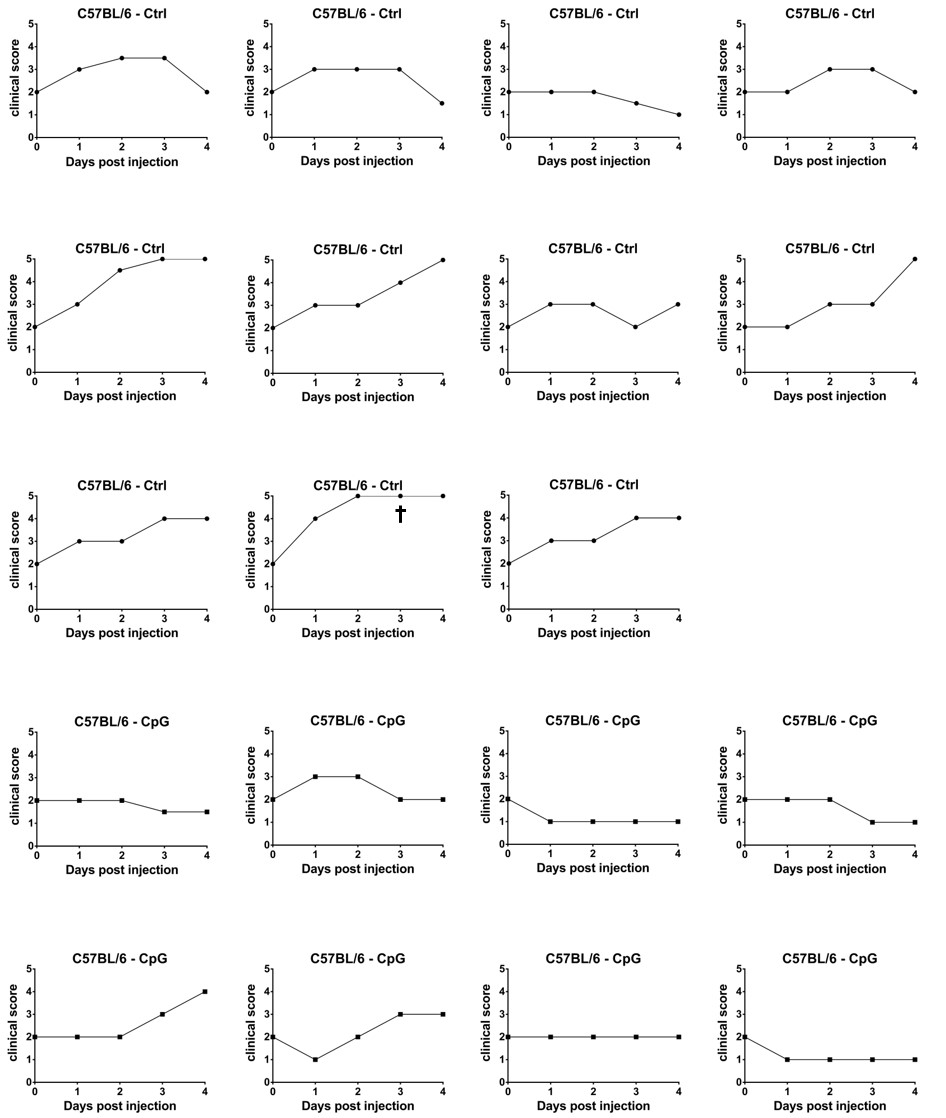

Supplement: Supplementary Figure 1 — Clinical scores of individual C57BL/6 mice with EAE that received CpG. Individual clinical scores of mice with EAE treated with CpG or vehicle (Ctrl). C57BL/6 mice were immunized with MOG35-55. At first sign of disease (day 0), mice received intrathecal injection of CpG or vehicle. A cross indicates the timepoint where a mouse was euthanized. [file Image_1.jpeg]

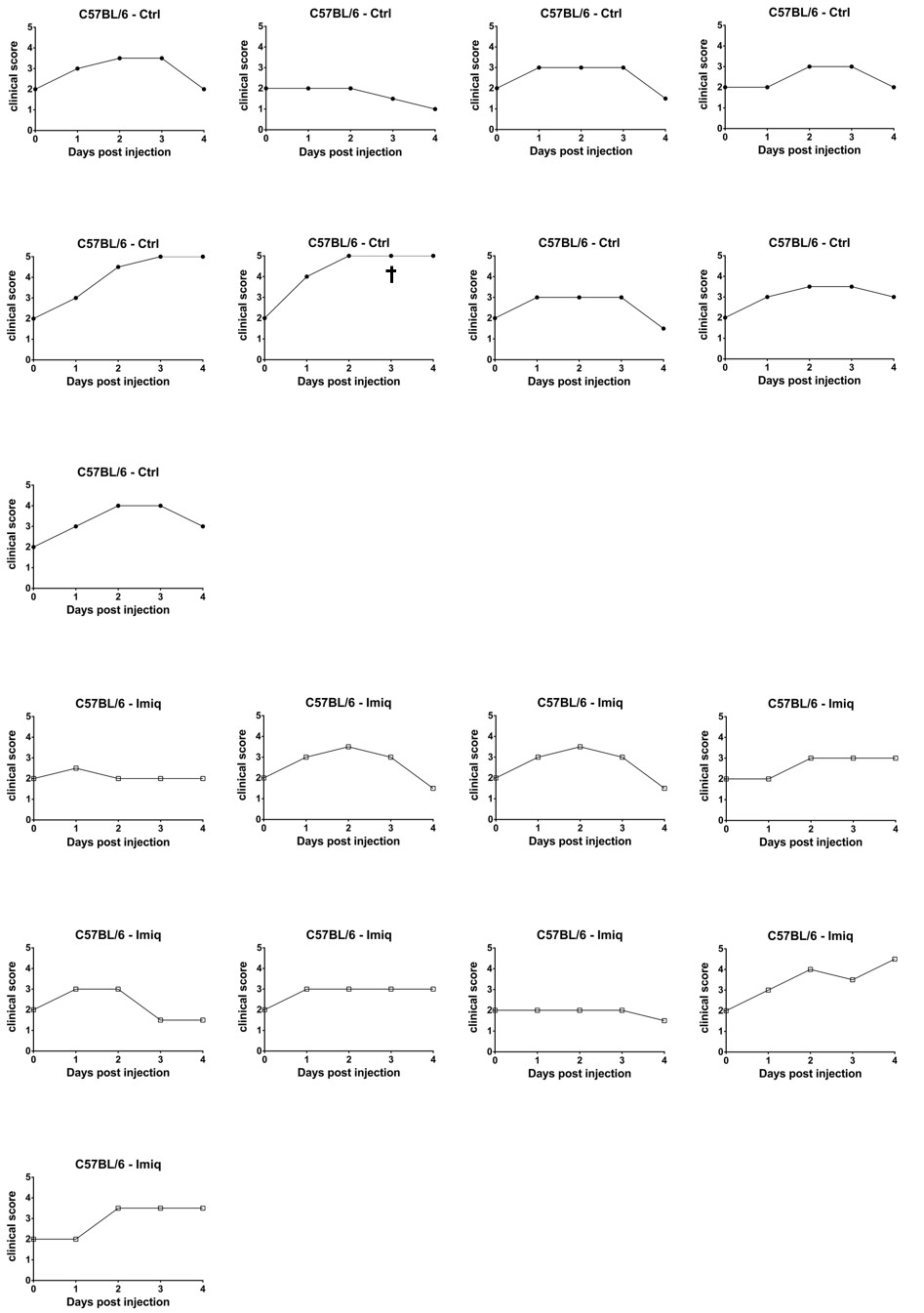

Supplement: Supplementary Figure 2 — Clinical scores of individual C57BL/6 mice with EAE that received Imiquimod. Individual clinical scores of mice with EAE treated with Imiquimod (Imiq) or vehicle (Ctrl). C57BL/6 mice were immunized with MOG35-55. At first sign of disease (day 0), mice received intrathecal injection of Imiquimod or vehicle. A cross indicates the timepoint where a mouse was euthanized. [file Image_2.jpeg]

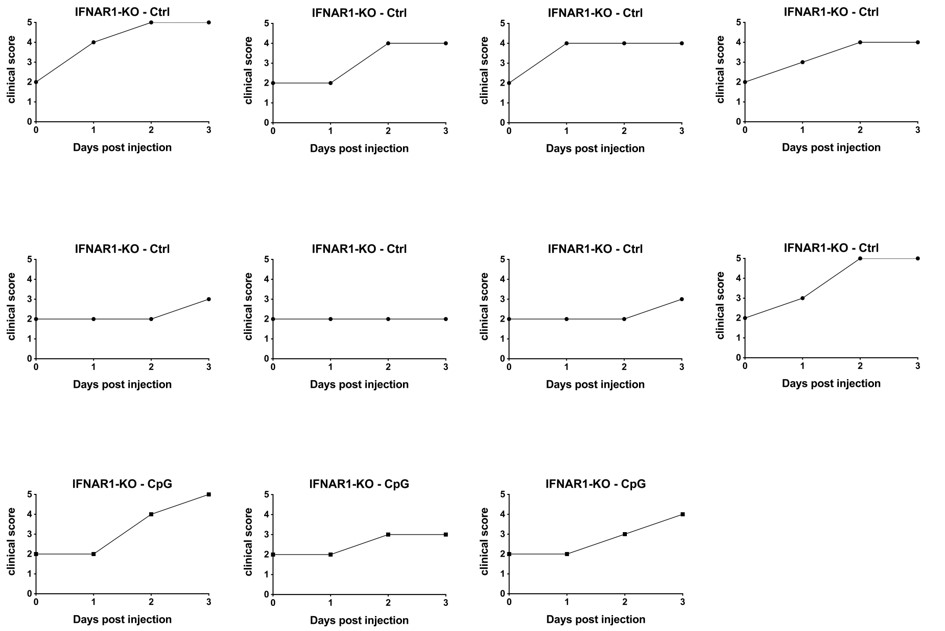

Supplement: Supplementary Figure 3 — Clinical scores of individual IFNAR1-KO mice with EAE that received CpG. Individual clinical scores of mice with EAE treated with CpG or vehicle (Ctrl). C57BL/6 mice were immunized with MOG35-55. At first sign of disease (day 0), mice received intrathecal injection of CpG or vehicle. [file Image_3.jpeg]

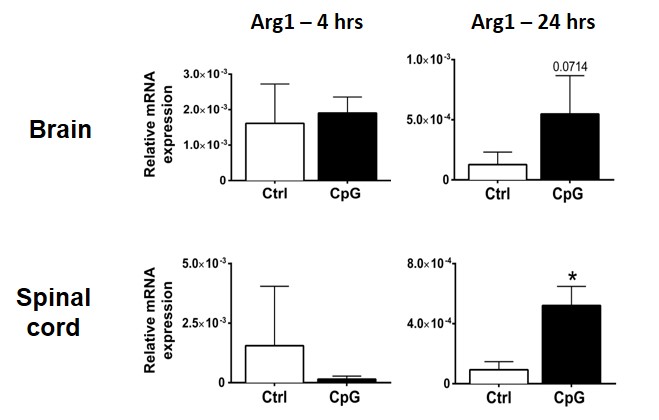

Supplement: Supplementary Figure 4 — Intrathecal CpG induces Arginase1. Brains and spinal cords were analyzed 4 and 24 h post intrathecal injection of CpG and Vehicle (Ctrl) into healthy C57BL/6 mice. Bar graphs show mRNA levels of Arginase1 (Arg1) (n = 3–5 per group). Results were analyzed using the two-tailed Mann–Whitney U-test. Data are presented as means ± SD. ∗p < 0.05. [file Image_4.jpeg]
